# 1358. Persistence of Omicron-Related Long COVID: Examining Prevalence and Impact at 12 Months Post-Acute Infection

**DOI:** 10.1093/ofid/ofad500.1195

**Published:** 2023-11-27

**Authors:** Yoonjung Kim, Shin-Woo Kim, Hyun-Ha Chang, Sohyun Bae

**Affiliations:** Kyungpook national university hospital, Daegu, Taegu-jikhalsi, Republic of Korea; School of Medicine, Kyungpook National University, Daegu, Taegu-jikhalsi, Republic of Korea; Division of Infectious Diseases, Department of Internal Medicine, School of Medicine, Kyungpook National University, Daegu, Korea, Daegu, Taegu-jikhalsi, Republic of Korea; Division of Infectious Diseases, Department of Internal Medicine, Kyungpook National University Hospital, School of Medicine, Kyungpook National University, Daegu, Republic of Korea, Taegu, Taegu-jikhalsi, Republic of Korea

## Abstract

**Background:**

As the COVID-19 pandemic continues, there is a growing interest in the long-term effects that follow COVID-19 infection, commonly known as long COVID. However, there is limited research on the long-term effects of the Omicron variant. Therefore, this prospective cohort study aimed to investigate the clinical characteristics of long COVID symptoms related to Omicron infection 12 months after the initial acute infection.

**Methods:**

The study included 94 adult patients diagnosed with COVID-19 between February 1, 2022, and March 2, 2022. Participants were scheduled to visit the study hospital four times, at 3, 6, 9, and 12 months after the acute COVID-19 infection, in order to assess their symptoms, quality of life, and mental health. The study investigated the clinical characteristics of the patients, self-reported long COVID symptoms, and measured quality of life using the EuroQol 5-dimension 5-level (EQ-5D-5L) index.

**Results:**

Out of the 94 respondents, 80 (85.1%) completed the study visits. Among them, 63 (78.8%) were female, and the median age was 36.0 [27.5; 48.5]. Long COVID symptoms still affected 42 (52.5%) patients 12 months after the acute infection. Four (5.0%) patients were diagnosed with new diseases following their COVID-19 infection. The most commonly reported long COVID symptoms after 12 months were fatigue, concentration difficulties, and amnesia (Figure 1). Insomnia and depression showed a gradual improvement in symptoms over time (Figure 2). Although the EQ-5D-5L index for the anxiety/depression dimension improved over time, it still affected 15.0% of the respondents (Figure 3).

**Figure 1.**
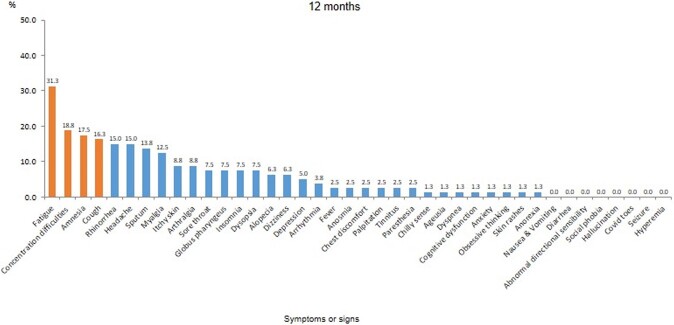
Distribution of 40 symptoms or signs at 12 months after acute COVID-19 infection

**Figure 2.**
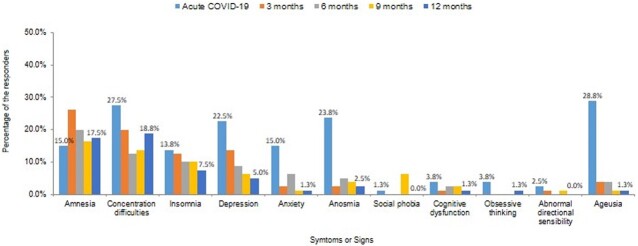
Prevalence difference of long COVID over time: acute COVID-19 period, 3, 6, 9, and 12 months from acute COVID-19 infection

**Figure 3.**
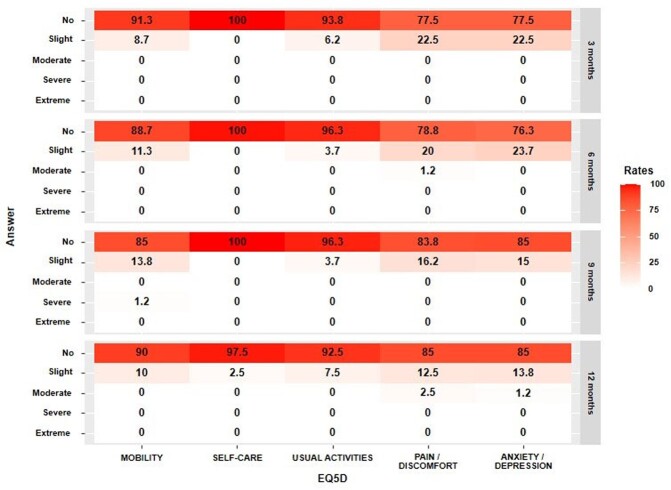
Assessment of quality of life (EQ-5D) at 3, 6, 9 and 12 months after acute COVID-19 infection

**Conclusion:**

This study highlights that neuropsychological symptoms may persist for a long time, even after Omicron variant infection. The study suggests that these symptoms can continue to be problematic, despite changes in the virus variant.

**Disclosures:**

**All Authors**: No reported disclosures

